# Diseases Admitted under the Care of the Physicians of the Westminster Hospital, from the 27th of April to the 24th of June, 1801

**Published:** 1801-07

**Authors:** 


*Difeafes admitted under the Care of the Phyficians of the Weftminfter Hofpital, from the 27th of April to the 24th of June,* 1801.

| | No. of Cafes. | | No. of Cafes. |
|---|---|---|---|
| Continued Fever | 31 | Hydrothorax | 3 |
| Intermittent | 1 | Hyfteria | 2 |
| Pleuritis | 7 | Leucorrhœa | 3 |
| Hepatitis | 1 | Lithiafis | 1 |
| Eryfipelas | 1 | Lumbago | 1 |
| Variola | 2 | Menorrhagia | 3 |
| Amenorrhœa | 4 | Obftipatio | 2 |
| Anafarca | 7 | Ophthalmia | 1 |
| Afcites | 4 | Paralyfis | 2 |
| Afthenia | 8 | Phthifis | 2 |
| Catarrh | 5 | Pleurodyne | 1 |
| Cephalæa | 4 | Podagra | 1 |
| Cholera | 4 | Rheumatifm | 14 |
| Colic | 2 | Scabies | 3 |
| Diarrhœa | 6 | Sciatica | 2 |
| Dyfpepfia | 4 | Struma | 2 |
| Dyfuria | 4 | Tabes | 3 |
| Enterodynia | 6 | Tuffis | 12 |
| Erythema | 1 | Vermes | 1 |
| Gaftrodynia | 6 | Vertigo | 3 |
| Hæmorrhois | 1 | | |

The unufual frequency of pleuritic and rheumatic affections, during the laft two months, appears to have arifen from the prevalence of cold eaft and north-eaft winds; but in what manner thefe winds affect the *human* frame, independent of temperature, we cannot at prefent determine.

The Works of Hippocrates on Air, Water, and Situation, abundantly convinces us that thefe circumftances had attracted confiderable attention in thofe times of almoft favage health. The valetudinarian ftate, induced by modern luxury, in diet, clothing, clofe rooms, down beds, and late hours, renders our bodies far more fufceptible of noxious impreffions from without, than the predeceffors and contemporaries of Hippocrates were. On this account it is our duty more particularly to endeavour to inveftigate the nature of thofe impregnations which render the air of certain fituations lefs falubrious than that of others. When it was difcovered that common atmofpherical air contained about 21 parts of vital air, or oxygen, and 79 of azote, or nitrogen, in 100 by meafure; it was haftily concluded by phyfiologifts, that we had acquired a power of afcertaining the comparative falubrity of atmofpherical air, during different winds, and in different places. For it was believed that the falubrity was proportional to the quantity of oxygen, and that this quantity admitted of confiderable variation in different fituations. It was however foon found, by the experiments of Dr. Prieftley and Mr. Cavendifh, and fince confirmed by many others, that the air of open feas, or the tops of high mountains, does not differ confiderably, in the proportion of thofe conftituents, from that of the ftreets of London. But when we fay the ftreets of London, we do not mean to include the rooms, theatres, exhibitions, &c. where either refpiration or combuftion, or both, are going on. This fource of contaminated air is, however, by no means confined to the metropolis; for where-ever well-jointed fafhes, double doors, double windows, or any other means of excluding frefh fupplies of air from inhabited rooms are employed, there a deterioration of it muft take place. The means of analyzing and correcting the water we ufe, are generally known; but the beft means of afcertaining the proportion of oxygen contained in the air we breathe, is known only to a few chemifts. We fhall, therefore, prefent our readers with Mr. Davy's new Eudiometer.—

"The dependance (he fays) of the health and exiftence of animals upon a peculiar ftate of the atmofphere, and the relations of this ftate to proceffes connected with the moft effential wants of life, have given intereft and importance to inquiries concerning the compofition and properties of atmofpheric air.

"This elaftic fluid has been long known to confift chiefly of oxygen and nitrogen, mingled together, or in a ftate of loofe combination, and holding in folution water.

"A va-

" A variety of proceſſes have been inſtituted, with the view of determining the relative proportion of the two gaſes, but moſt of them have involved ſources of inaccuracy; and lately all, except two (the ſlow combuſtion of phoſphorus, and the action of liquid ſulphurets) have been generally abandoned.

Both phoſphorus and ſolution of ſulphuret of potaſh abſorb the whole of the oxygen of atmoſpheric air at common temperatures, and they do not materially alter the volume, or the properties of the reſidual nitrogen; but their operation is extremely ſlow; and in many caſes it is difficult to aſcertain the period at which the experiment is completed.

" I have lately employed as an eudiometrical ſubſtance the ſolution of green muriat, or ſulphat, of iron, impregnated with nitrous gas; and I have found that it is in ſome reſpects ſuperior to many of the bodies heretofore uſed, as it rapidly condenſes oxygen without acting upon nitrogen; and requires for its application only a very ſimple and a very portable apparatus.

" This fluid is made by tranſmitting nitrous gas through green muriat, or ſulphat, of iron, diſſolved to ſaturation in water.* As the gas is abſorbed, the ſolution becomes of a deep olive brown, and when the impregnation is completed it appears opaque and almoſt black. The proceſs is apparently owing to a ſimple elective attraction; in no caſe is the gas decompoſed; and under the exhauſted receiver it aſſumes its elaſtic form, leaving the fluid with which it was combined unaltered in its properties.

" The inſtruments neceſſary for aſcertaining the compoſition of the atmoſphere, by means of impregnated ſolutions, conſiſt ſimply of a ſmall graduated tube, having its capacity divided into one hundred parts, and greateſt at the open end; and of a veſſel for containing the fluid.

" The tube, after being filled with the air to be examined, is introduced into the ſolution; and, that the action may be more rapid, gently moved from a perpendicular towards a horizontal poſition. Under theſe circumſtances the air is rapidly diminiſhed; and, in conſequence of the dark colour of the fluid, it is eaſy to diſcover the quantity of abſorption. In a few minutes the experiment is completed, and the whole of the oxygen condenſed by the nitrous gas in the ſolution in the form of nitrous acid.

In

* Dr. Prieſtley firſt obſerved this proceſs: for a particular account of it ſee Reſearches, Chemical and Philoſophical, page 152. Johnſon.

"In all eudiometrical proceſſes with impregnated ſolutions, the period at which the diminution is at a ſtand muſt be accurately obſerved; for, ſhortly after this period, the volume of the reſidual gas begins to be a little increaſed, and, after ſome hours, it will often fill a ſpace greater by ſeveral of the hundred parts on the ſcale of the tube, than that which it occupied at the maximum of abſorption.

"This circumſtance depends upon the ſlow decompoſition of the nitrous acid (formed during the experiment), by the green oxide of iron, and the conſequent production of a ſmall quantity of aëriform fluid (chiefly nitrous gas);* which, having no affinity for the red muriate, or ſulphate, of iron produced, is gradually evolved, and mingled with the reſidual nitrogen.

"The impregnated ſolution with green muriate is more rapid in its operation than the ſolution with green ſulphate. In caſes when theſe ſalts cannot be obtained in a ſtate of abſolute purity, the common or mixed ſulphate of iron may be employed. One cubic inch of moderately ſtrong impregnated ſolution is capable of abſorbing five or ſix cubic inches of oxygen, in common proceſſes; but the ſame quantity muſt never be employed for more than one experiment.

"A number of comparative experiments, made on the conſtitution of the atmoſphere at the Hotwells, Briſtol, in July, Auguſt, and September, 1800, with phoſphorus, ſulphurets of alkalies, and impregnated ſolution, demonſtrated the accuracy of the proceſſes in which the laſt ſubſtance was properly employed. The diminutions given by the ſulphurets were indeed always greater by a minute quantity than thoſe produced by phoſphorus and impregnated ſolutions: but the reaſon of this will be obvious to thoſe who have ſtudied the ſubject of Eudiometry. In no inſtance was it found that 100 parts in a volume of air contained more than 21 of oxygen: *and the variations connected with different winds, and different ſtates of temperature, moiſture, &c. were too ſmall, and too often related to accidental circumſtances, to be accurately noticed.*

"In analyſing the atmoſphere in different places, by means of impregnated ſolutions, I have never been able to aſcertain any notable difference in the proportions of its conſtituent parts. Air, collected on the ſea at the mouth of the Severn, on

* The decompoſition of nitrous acid, by ſolutions containing oxide of iron, at its minimum of oxidation, is a very complex proceſs. The green oxide, during its converſion into red oxide, not only decompoſes the acid, but likewiſe acts upon the water of the ſolution; and ammoniac is ſometimes formed, and ſmall portions of nitrous oxide and nitrogen evolved with the nitrous gas.

on October the 3d, 1800, which must have passed over much of the Atlantic, as the wind was blowing strong from the west, was found to contain 21 per cent. of oxygen in volume; and this was nearly the proportion in air sent from the coast of Guinea to Dr. Beddoes, by two surgeons of Liverpool.

"If we compare these results with the results gained more than twenty years ago by Mr. Cavendish, from experiments on the composition of atmospherical air, made at London and Kensington; considering, at the same time, the researches of Berthollet in Egypt and at Paris, and those of Marti in Spain, we shall find strong reasons for concluding, that the atmosphere, in all places exposed to the influence of the winds, contains very nearly the same proportions of oxygen and nitrogen: a circumstance of great importance; for, by teaching us that the different degrees of salubrity of air do not depend upon differences in the quantities of its principal constituent parts, it ought to induce us to institute researches concerning the different substances capable of being dissolved or suspended in air, which are noxious to the human constitution; particularly as an accurate knowledge of their nature and properties would probably enable us, in a great measure, to guard against, or destroy, their baneful effects."

The most obvious impregnation of the atmosphere is that with distilled water; and many people have taken up an opinion, that, independent of *miasmata*, the only noxious qualities of the atmosphere depend on its moisture, or the quantity of water diffused through it, when the thermometer is near 32°, or, as they express it, "when cold and moisture are combined;" but we shall endeavour to shew, on some future occasion, that this opinion is not well founded.

